# TGF-β1-induced HSP47 regulates extracellular matrix accumulation via Smad2/3 signaling pathways in nasal fibroblasts

**DOI:** 10.1038/s41598-019-52064-1

**Published:** 2019-10-29

**Authors:** Hae-Ji Kim, Joo-Hoo Park, Jae-Min Shin, Hyun-Woo Yang, Heung-Man Lee, Il-Ho Park

**Affiliations:** 1 Upper Airway Chronic inflammatory Diseases Laboratory, Korea University, College of Medicine, Seoul, Korea; 2 Medical Devices Clinical Trials Laboratory, Korea University, College of Medicine, Seoul, Korea; 3 IVD Support Center Korea University, Korea University, College of Medicine, Seoul, Korea; 4 Department of Otorhinolaryngology-Head and Neck Surgery, Korea University, College of Medicine, Seoul, Korea

**Keywords:** Mechanisms of disease, Extracellular signalling molecules

## Abstract

HSP47 is required for the production of collagen and serves an important role in tissue remodeling, a pathophysiologic mechanism of chronic rhinosinusitis (CRS). We investigated the relationship between HSP47 expression and tissue remodeling in CRS. We also determined the underlying molecular mechanisms of TGF-β1-induced HSP47 and extracellular matrix (ECM) production in nasal fibroblasts. HSP47, α-SMA, fibronectin, and collagen type I expression levels were measured using real-time PCR, western blotting, and immunofluorescence staining. Fibroblast migration was analyzed using scratch and transwell migration assays. Contractile activity was measured with a collagen gel contraction assay. HSP47 is increased in patients with CRS without nasal polyps. TGF-β1 induced HSP47 expression in nasal fibroblasts. Myofibroblast differentiation and ECM production, which are induced by TGF-β1, were inhibited by *siHSP47*. We also confirmed that the Smad2/3 signaling pathway is involved in TGF-β1-induced HSP47 expression in nasal fibroblasts. In a functional assay, TGF-β1-enhanced migration and contraction ability were inhibited by HSP47 knockout. Glucocorticoid reversed the stimulatory effects of TGF-β1 on HSP47 expression and ECM production in nasal fibroblasts and *ex vivo* organ cultures. HSP47 expression is involved in TGF-β1-induced myofibroblast differentiation and ECM production through the Smad2/3 signaling pathway, which might contribute to tissue remodeling in chronic rhinosinusitis.

## Introduction

Chronic rhinosinusitis (CRS), one of the most frequent chronic diseases in humans, is defined as sinonasal inflammation with symptoms of nasal discharge or postnasal drip, nasal congestion, sinus pain or pressure, and anosmia or hyposmia, lasting for at least 12 weeks^1^. CRS affects nearly 15% of the world’s population and has an important socio-economic impact^2^. Its effect on the quality of life of patients is significant; studies have shown that the quality of life of patients with CRS is similar or worse as compared to patients with other chronic diseases such as congestive heart failure, chronic obstructive pulmonary disease, and back pain^3^. CRS is not a disease caused by a single factor but a multifactorial disorder that involves interplay among environmental insults, infectious loading, and genetic predisposition in its pathogenesis. Based on differential inflammatory and remodeling patterns, CRS can be divided into chronic rhinosinusitis without nasal polyps (CRSsNP) and chronic rhinosinusitis with nasal polyp formation (CRSwNP)^4^.

Tissue remodeling, a dynamic process involving the production and degradation of the extracellular matrix (ECM), is important in the process of wound healing for all organs of the human body, and this sometimes involves pathological tissue regeneration^5^. Chronic airway diseases such as asthma and CRS stimulate mechanisms of airway healing and repair that can lead to progressive, potentially irreversible, tissue destruction. Airway remodeling is clearly present in chronic sinus disease^6^, and ECM accumulation is an important feature of such remodeling. Fibroblasts play a role in structurally maintaining tissue morphology and inducing ECM production. During this process, fibroblasts develop into myofibroblasts that express α-smooth muscle actin (α*-*SMA), subsequently resulting in increased deposition of ECM components, such as fibronectins and collagen type I^7^. Myofibroblast differentiation and ECM production are acknowledged as important phenomena for understanding the pathogenesis of CRS^8^.

Heat shock protein 47 (HSP47) is a collagen-specific molecular chaperone required for molecular maturation of various types of collagens during procollagen folding^9^. HSP47, believed to be an important modulator of various physiological and pathological processes, is a 47-kDa stress protein localized in the endoplasmic reticulum of cells synthesizing collagens^10^. It is well known that HSP47 is expressed during the process of fibrosis, particularly in and around fibrotic lesions. Myofibroblasts, types of HSP47-positive cells, are proposed to play a key role in the synthesis, deposition, and remodeling of the ECM in nasal fibrosis in human patients and animal models^11^.

We hypothesized that fibrosis observed during CRS is related to HSP47. We presumed that HSP47 serves a role in fibrotic features related to CRS pathogenesis, such as fibroblast activation and ECM accumulation. Here, we investigate whether HSP47 is related to components of CRSsNPs and determine which cytokine induces HSP47 expression. We also determine the effect of glucocorticoids on TGF-β1-induced HSP47 and ECM production, cell migration, invasion, and collagen contraction in nasal fibroblasts.

## Methods

### Human subjects

Sinonasal and nasal polyp tissues were obtained from 37 patients (12 male and 25 female; mean age 45.2 ± 5.9 years). Normal uncinate process (UP) tissues were obtained during rhinoplasty. CRS diagnosis was based on historical, endoscopic, and radiographic criteria, as well as CT findings of sinuses according to the 2012 European Position Paper on Rhinosinusitis and Nasal Polyps (EPOS) guidelines^12^. Nasal polyp tissues were obtained from the middle meatus region at the beginning of endoscopic surgery in CRSwNP patients. All the patient agreed to the informed consent form and the Korea University Medical Center Institutional Review Board approved of the study (KUGH12041-001). Written informed consent was obtained from all subjects, and this study was conducted according to the principles of the Declaration of Helsinki. No patients had taken oral steroids, non-steroidal anti-inflammatory drugs, antihistamines, or antibiotics for at least 4 weeks. Clinical characteristics of patients are summarized in Table SI.

### Nasal fibroblast culture

Six patients (2 male and 4 female; mean age 40.3 ± 4.0) with inferior turbinate tissue were recruited from the Department of Otorhinolaryngology, Korea University Medical Center, and inferior turbinate tissues were obtained during surgical procedures. All patients were nonsmokers and had not been treated with oral antibiotics or antihistamines for at least 4 weeks before surgery. This study was approved by the Korea University Medical Center Institutional Review Board. Inferior turbinate tissue was cut into 0.3 to 0.5 mm fragments, re-suspended in in Dulbecco’s modified Eagle’s medium (DMEM; Invitrogen, Grand Island, NY) containing collagenase (500 U/mL; Sigma), hyaluronidase (30 U/mL; Sigma), and DNAse (10 U/mL; Sigma), 100 g/mL of penicillin, and 100 g/mL of streptomycin (Invitrogen) for enzymatic digestion. After 2 hours incubation in 5% CO_2_ at 37 °C, the cells were collected by centrifugation, washed twice, resuspended in DMEM containing 10% (v/v) heat-inactivated fetal bovine serum and antibiotics, 100 g/mL of penicillin, and 100 g/mL of streptomycin. Cells were allowed to attach the culture plate for 4 days. The medium was changed Non-adherent cells were removed by changing the medium. Fibroblasts were detached with trypsin–EDTA solution (Invitrogen). After the cells were washed, the cells were resuspended in medium and used for subsequent experiments. Fibroblast purity was 99% and used for nasal fibroblasts.

### Real-time PCR

We extracted RNA using TRIzol reagent (Invitrogen), and a Maxime RT PreMix cDNA kit (Intron, Biotechnology) was used to generate cDNA. Real-time PCR was performed with Quantstudio3 (Applied Biosystems, Foster City, CA) using Power SYBR Green PCR Master Mix (Applied Biosystems). Relative gene expression was evaluated by analyzing real-time PCR data using the 2^−ΔΔCt^ (2DDCt) method. Expression levels of the target mRNAs were normalized to *GAPDH*. Experiments were repeated at least three times. Forward and reverse primers used for PCR are shown in Supplementary Table S2.

### Western blot analysis

Nasal fibroblast cells were lysed with RIPA buffer (Cell Signaling Technology, Danvers, MA, USA). The lysate was centrifuged and quantitated using Bradford assay reagent (Bio-Rad, Hercules, CA, USA) following the manufacturer’s protocol. Proteins were separated by 10% sodium dodecyl sulfate polyacrylamide gel electrophoresis and transferred onto polyvinyl difluoride membranes (Millipore Inc., Billerica, MA, USA). Membranes were blocked with 5% skim milk for 1 h at room temperature. Western blot analysis was performed using the following antibodies: HSP47, α-SMA, fibronectin, GAPDH (Santa Cruz Biotechnology, Inc., Santa Cruz, CA, USA), collagen type I, phospho-Smad2/3, total-Smad2/3 (Cell Signaling Technology, Danvers, MA, USA). Membranes were washed three times for 5 min each and incubated with HRP-conjugated anti-mouse or anti-rabbit antibodies (Vector Laboratories, Burlingame, CA, USA) for 2 h. Blots were visualized with the ECL system (Pierce, Rockford, IL). Images were analyzed using ImageJ software (NIH, Rockville, MD, USA)^13^. Protein expression was normalized to β-actin or the total protein of each phosphorylated protein.

### Short interfering RNA transfection

To analyze the mRNA and protein expression levels of Smad2/3, HSP47, and ECM under HSP47 knockdown conditions, nasal fibroblasts were transfected with short interfering (si)RNA directed against *HSP47* (1135737, Bioneer, Daejeon, Korea) or negative control siRNA (SN-1013, Bioneer) according to the manufacturer’s instructions. Lipofectamine transfection reagent and *HSP47* siRNA (100 nM) were mixed in Opti-MEM cell culture medium, and the cells were incubated in the mixture. Transfected cells were treated with TGF-β1 and incubated at 37 °C.

### Immunofluorescence staining

Nasal fibroblasts, sinonasal tissue, and *ex vivo* organ cultures were fixed with 4% paraformaldehyde for 30 min and permeabilized with 0.01% Triton X-100 (Sigma-Aldrich, St. Louis, MO, USA). Cells and tissue were blocked with 3% bovine serum albumin for 1 h. Nasal fibroblasts and tissue were incubated with primary antibodies anti-HSP47 (1:1,000), anti-α SMA (1:1,000), anti-fibronectin (1:1,000), or anti-collagen type 1 (1:500) overnight at 4 °C. Nasal fibroblasts and tissue were then incubated with anti-mouse Alexa 488 (Invitrogen) or anti-rabbit Alexa 555 (Invitrogen) secondary antibodies for 1 h. Counterstaining was performed using 4′-6-diamidino-2-phenylindole (DAPI, Sigma-Aldrich). Image acquisition and processing were performed using a confocal laser scanning microscope LSM700 (Zeiss, Oberkochen, Germany). Expression of HSP47, ECM and p-Smad2/3 were determined by mean fluorescence intensity and Manders’ overlap coefficient analysis using image software^14^.

### Wound scratch assay

After nasal fibroblasts cultured in 6-well dishes reached 80% confluence, they were scratched with a pipette tip. Scratched cells were immediately rinsed with phosphate buffered saline (PBS) and DMEM medium containing 10% (v/v) heat-inactivated FBS (Invitrogen), 10,000 unit/mL penicillin, and 10,000 μg/mL streptomycin (Invitrogen). Cells were incubated with TGF-β1 for 48 h. Migrated nasal fibroblasts were stained using Diff-Quik stain (Sysmex, Kobe, Japan). Images were produced under a microscope (Olympus BX51; Olympus, Tokyo, Japan). The cell migration rate is presented as the ratio of the migration distance in test cells relative to that in control cells.

### Transwell migration assay

Nasal fibroblasts were seeded into transwell inserts with 8.0 μm pores (Sigma-Aldrich) on a 24-well plate. Serum-free DMEM was added to the bottom chamber. After 48 h, non-invasive cells were removed from the upper chamber, and invasive cells were stained with Diff-Quik stain (Sysmex). Then, migrated cells on the lower wall surface were fixed with methanol and stained with Diff-Quick stain (Sysmex) for 10 min. The number of cells invading the membrane was counted from 5 randomly selected visual fields using an inverted microscope (Olympus BX51; Olympus) at ×200 magnification.

### Collagen gel contraction assay

Nasal fibroblasts (3 × 10^5^) were mixed with type I collagen solution, serum-free DMEM, and reconstituting buffer (260 mM NaHCO_3_, 200 mM HEPES, and 50 mM NaOH), and the cell suspensions were mixed carefully on ice at a ratio of 7:2:1:1. Then, 500 μL of the reconstituted collagen mixture was placed in each well of a 24-well tissue culture plate, and the gel was allowed to solidify at room temperature for 30 min. After the gels had solidified, 600 μL of culture media was added to each well along with TGF-β1. The gels were then incubated at 37 °C in a 5% CO_2_ atmosphere for 3 days. The area of each gel was measured using an Image J analyzer (NIH). Data are expressed as the percentage of the measured area relative to the initial gel area.

### *Ex vivo* organ culture

Nasal inferior turbinate tissues were cut into 3–4-mm^3^ pieces, rinsed three times with PBS, and cultured in DMEM supplemented with 2% FBS (Invitrogen), 1% 10,000 unit/mL penicillin, and 1% 10,000 μg/mL streptomycin (Invitrogen). Nasal inferior turbinate tissues were placed on 3.0-μm pore size Transwell permeable supports (Corning Costar, Corning, NY, USA), with the mucosa side facing up and the submucosa side facing down. Nasal inferior turbinate tissues were pretreated with or without dexamethasone (5 μM) or fluticasone propionate (5 μM) and subsequently treated with TGF-β1 (1 ng/mL) for 72 h.

## Results

### HSP47 is increased in patients with CRSsNP

**Figure 1:**
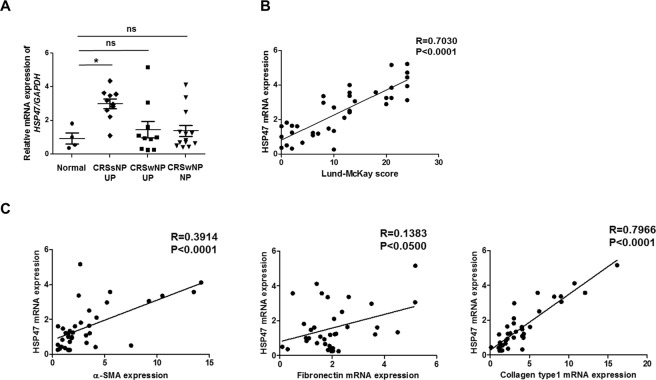
HSP47 expression in nasal tissues. (**A**) HSP47 mRNA expression analyzed using real-time PCR. Tissue from CRSsNP patients showed significantly higher level of HSP47 expression relative to nasal mucosa from the normal control (p = 0.0067) and patients with CRSwNP (2.30 ± 0.75; p = 0.048, Kruskal-Wallis 1-way ANOVA). (**B**) The correlation between Lund-McKay score and HSP47 expression in normal control and CRS patients (R square = 0.2391). (**C**) The correlations between HSP47 expression and extracellular matrix-related makers in the normal control (n = 4) and CRS patients (n = 33). Data are expressed as the mean ± SEM of three independent experiments. *p < 0.05 vs. Normal.

HSP47, a collagen chaperone protein, modulates ECM deposition. Overexpression of HSP47 contributes to inflammatory airway conditions. To confirm the correlation between HSP47 and CRS, real-time PCR was performed using nasal tissues from each group (Control UP, n = 4; CRSsNP-UP, n = 10; CRSwNP-UP, n = 10; CRSwNP-NP, n = 13). We analyzed whether it was possible to distinguish between patients with CRSwNP or CRSsNP based on severity using the Rhinosinusitis Lund-McKay CT scores. *HSP47* mRNA levels were found to be significantly elevated in tissue from patients with CRSsNP-UP (2.996 ± 0.283) relative to the normal healthy control group (0.933 ± 0.324) (Fig. 1A). HSP47 expression was positively correlated with the Lund-Mackay CT score (Fig. 1B). Additionally, the expression of ECM components, such as collagen type 1, was also highly correlated with HSP47 expression (Fig. 1C).

### TGF-β1 induces HSP47 expression in nasal fibroblasts

**Figure 2:**
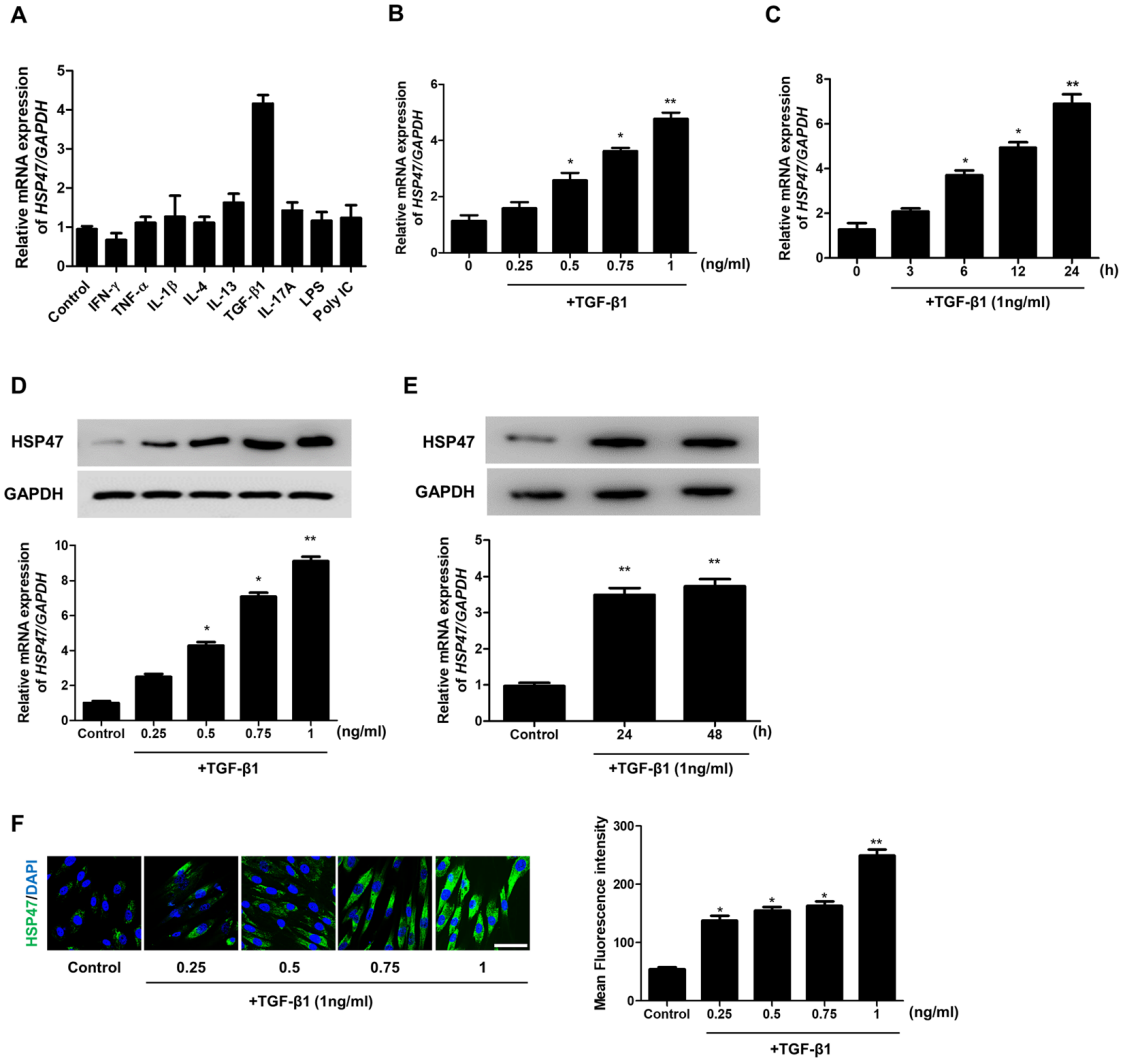
TGF-β1 induces HSP47 expression in nasal fibroblasts. (**A**) HSP47 mRNA expression was measured by real-time PCR in nasal fibroblast cells (n = 6) after stimulation with various TLR ligands (IFN-γ (100 ng/mL), TNF-α (50 ng/mL), IL-1β (10 ng/mL), IL-4 (10 ng/mL), IL-13 (10 ng/mL), TGF-β1 (1 ng/mL), IL-17A (10 ng/mL), LPS (10 ng/mL), and Poly IC (10 μg/mL) for 24 h. (**B**) Real-time PCR analyses revealed a dose-dependent increase in the mRNA expression of *HSP47* following treatment with TGF-β1. (**C**) Real-time PCR analyses of *HSP47* revealed time-dependent increases in mRNA expression following TGF-β1 treatment. (**D**) TGF-β1 treatment increased HSP47 protein expression in a dose-dependent manner. (**E**) TGF-β1 treatment also increased HSP47 protein expression in cells in a time-dependent manner. Nasal fibroblast cells were treated with TGF-β1, and HSP47 expression was detected by western blotting. (**F**) TGF-β1 increased HSP47 expression in a dose-dependent manner in nasal fibroblast cells. Immunofluorescence was detected using confocal laser scanning microscopy. For negative control, the cells were incubated with corresponding secondary antibodies after omitting the primary antibody. Representative fluorescein immunocytochemical staining showed HSP47 (green) and nuclear DAPI (blue). Scale bar = 50 μm. Data are expressed as the mean ± SEM of three independent experiments. *p < 0.05 vs. control; **p < 0.01 vs. control.

To determine whether various stimuli induce HSP47 expression in nasal fibroblasts, nasal fibroblasts were treated with various stimuli such as Th1 cytokines (IFN-γ and TNF-α), Th2 cytokines (IL-4 and IL-13), pro-inflammatory cytokine (TGF-β1), Th17 cytokine (IL-17A), Toll-like receptor ligands (LPS and poly[I:C]), and IL-1β. Among these cytokines, only TGF-β1 significantly increased *HSP47* mRNA levels (Fig. 2A). We evaluated the effect of TGF-β1 on the expression of *HSP47* mRNA and protein in dose- and time-dependent manners. TGF-β1 significantly increased *HSP47* mRNA and protein expression in a dose-dependent manner (Fig. 2B,D). TGF-β1 increased *HSP47* mRNA expression over time (Fig. 2C). TGF-β1 also increased HSP47 protein expression in 24 and 48 h (Fig. 2E). As shown by immunofluorescence observation, TGF-β1 increased HSP47 expression in nasal fibroblasts in a dose-dependent manner (Fig. 2F).

### *HSP47* (si)RNA inhibits TGF-β1-induced myofibroblast differentiation and extracellular matrix production in nasal fibroblasts

**Figure 3:**
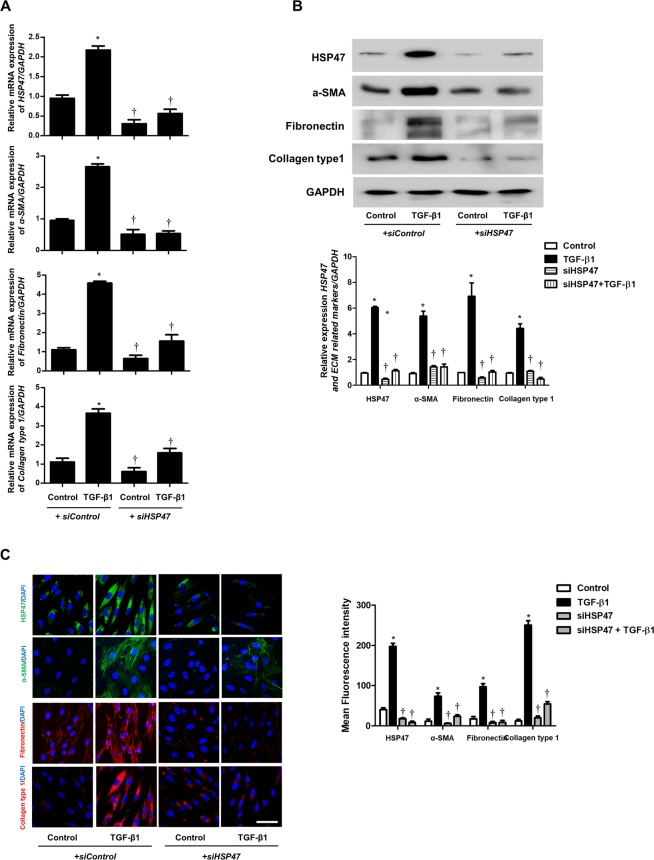
TGF-β1-induced myofibroblast extracellular matrix production in nasal fibroblasts is inhibited by *HSP47* (si) RNA. (**A**) Specific *HSP47* siRNAs were transfected before treatment with or without TGF-β1 for 24 h in nasal fibroblasts (n = 6). The mRNA levels of *HSP47* and ECM-related markers were verified by real-time PCR. (**B**) Specific *HSP47* siRNAs were transfected before treatment with or without TGF-β1 for 48 h. Protein levels of HSP47 and ECM-related markers were determined by western blotting. (**C**) Nasal fibroblasts transfected with *HSP47* siRNA were stimulated with or without TGF-β1 for 48 h. Immunofluorescence was detected using confocal laser scanning microscopy. For negative control, the cells were incubated with corresponding secondary antibodies after omitting the primary antibody Representative fluorescein immunocytochemical staining showed HSP47 (green), α-SMA (green), fibronectin (red), and collagen type 1 (red) with nuclear DAPI (blue). Scale bar = 50 μm. Data are expressed as the mean ± SEM of three independent experiments. *p < 0.05 vs. control; ^†^p < 0.05 vs. TGF-β1.

In nasal fibroblasts, TGF-β1 induced myofibroblast differentiation and ECM accumulation, which are involved with tissue remodeling. To determine whether HSP47 was related to TGF-β1-induced myofibroblast differentiation and ECM production in nasal fibroblasts, HSP47 was silenced by *siHSP47* transfection. Knockdown of *HSP47* with siRNA inhibited TGF-β1-induced HSP47, α-SMA, and ECM production in nasal fibroblasts (Fig. 3A,B). Based on immunocytochemical staining, the expression of HSP47 and ECM were inhibited by *siHSP47* in TGF-β1-stimulated fibroblasts (Fig. 3C).

### TGF-β1 induces HSP47 expression via the Smad2/3 pathway

**Figure 4:**
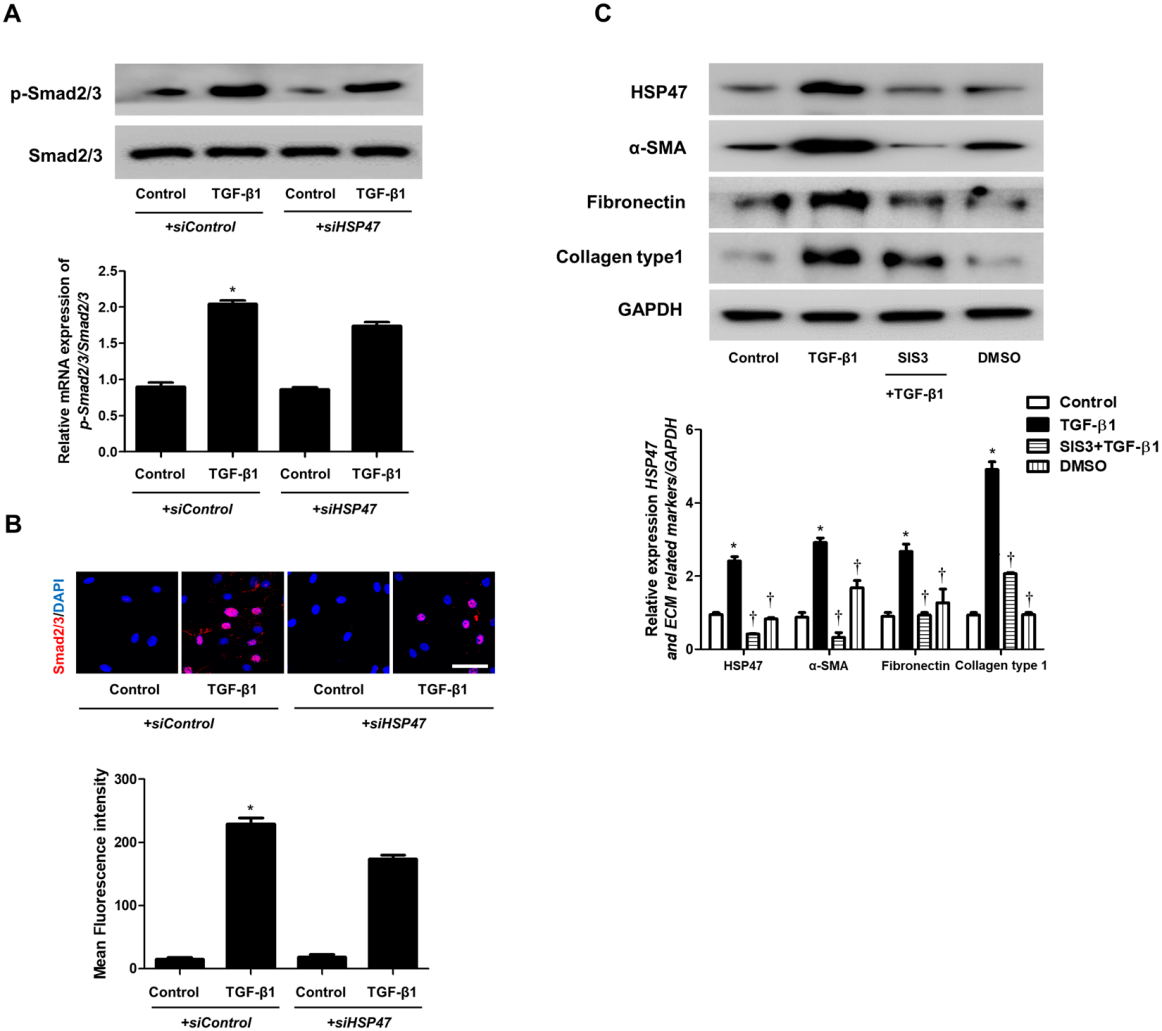
TGF-β1 induces Smad2/3 phosphorylation in nasal fibroblasts. (**A**) Phosphorylation of the Smad2/3 signaling pathway component p-Smad2/3 in nasal fibroblasts (n = 6) was detected by western blotting. (**B**) Immunocytochemistry was performed to confirm the translocation of Smad2/3. For negative control, the cells were incubated with corresponding secondary antibodies after omitting the primary antibody. Scale bar = 50 μm. (**C**) The Smad2/3 inhibitor SIS3 (Smad3-specific inhibitor, 3 μM) was used to pretreat cells for 1 h before treatment with TGF-β1 for 48 h. HSP and ECM-related markers were detected by western blot. Data are expressed as the mean ± SEM of three independent experiments. *p < 0.05 vs. control; ^†^p < 0.05 vs. TGF-β1.

Smad2/3, intracellular mediators of TGF-β1 signaling, are involved with TGF-β1-induced myofibroblast differentiation and ECM production in nasal fibroblasts. To determine the role of Smad2/3 signaling in TGF-β1-induced HSP47, α-SMA, fibronectin, and collagen type1 expression, fibroblasts were treated with a Smad2/3 inhibitor, SIS3. TGF-β1-induced phosphorylation of Smad2/3 was not inhibited by *siHSP47* transfection (Fig. 4A). TGF-β1-induced overexpression and translocation of Smad2/3 from the cytoplasm to the nucleus also were not affected by *siHSP47* transfection (Fig. 4B). Inhibition of Smad2/3 phosphorylation by SIS3 significantly down-regulated TGF-β1-stimulated production of HSP47, α-SMA, fibronectin, and collagen type 1 (Fig. 4C). These results indicate that HSP47 regulates TGF-β1-stimulated myofibroblast differentiation and ECM production through the Smad2/3 signaling pathway in nasal fibroblasts.

### Knockdown of HSP47 inhibits increased migration and contractile ability in TGF-β1-stimulated nasal fibroblasts

**Figure 5:**
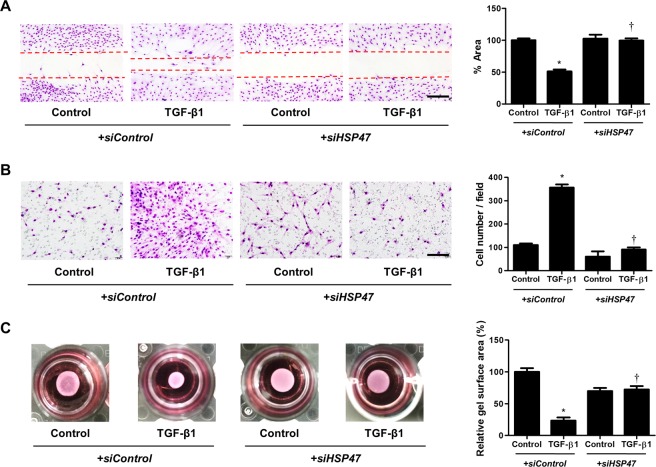
HSP47 induces cell migration and collagen contraction in nasal fibroblasts. (**A**) A wound scratch assay was used to confirm migration of nasal fibroblasts (n = 6). (**B**) Inhibitory effects of HSP47 siRNA on migration and invasion over a 48-h period were shown using a transwell migration assay. (**C**) Contractile activity was measured using a collagen gel contraction assay, and the contraction area was measured using an Image J analyzer. Scale bar = 20 μm. Data are expressed as the mean ± SEM of three independent experiments. *p < 0.05 vs. control; ^†^p < 0.05 vs. TGF-β1.

To confirm the effect of HSP47 on cell migration and collagen gel contractile ability, we performed wound scratch migration, transwell migration, and collagen gel contraction assays. Fibroblast migration was significantly increased by TGF-β1 treatment (51.040 ± 3.068) relative to the control group (100 ± 2.887). However, transfection of *siHSP47* inhibited TGF-β1-induced cell migration (101.153 ± 0.714) (Fig. 5A). In the Transwell migration assay, TGF-β1-induced migrated nasal fibroblasts (355.333 ± 14.438) were significantly suppressed by transfection of *siHSP47* (91.667 ± 4.910) (Fig. 5B). Transfection of *siHSP47* (73.159 ± 2.315) inhibited TGF-β1-induced collagen gel contraction (23.612 ± 4.659) (Fig. 5C).

### Glucocorticoid reversed the stimulatory effects TGF-β1 on HSP47 and ECM production in nasal fibroblasts

**Figure 6:**
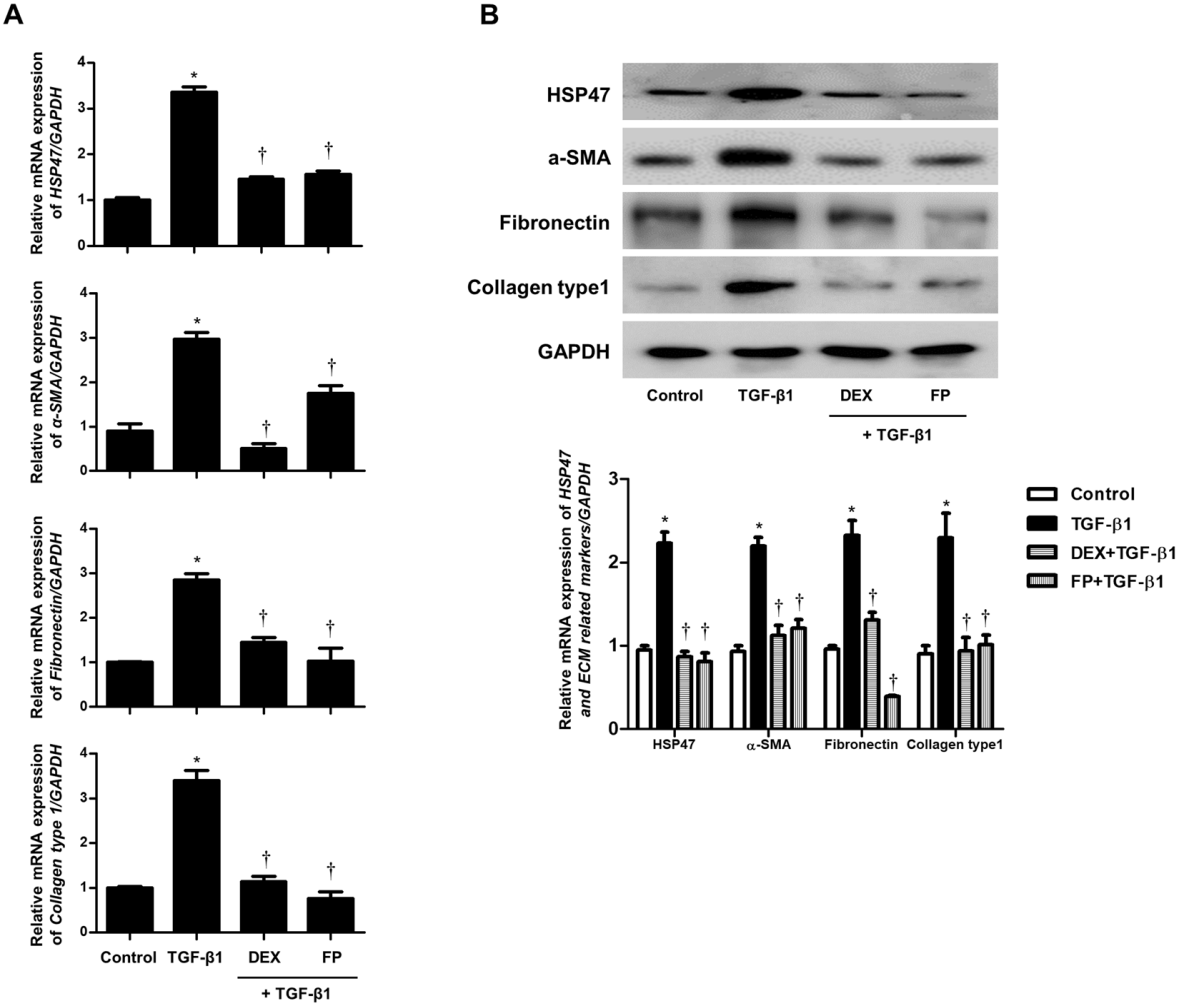
Glucocorticoids reduce TGF-β1-induced HSP47 and extracellular matrix expression in nasal fibroblasts. Cells were pretreated with or without dexamethasone (2.5 μM) and fluticasone propionate (2.5 μM) and then treated with TGF-β1 (1 ng/mL) in nasal fibroblast (n = 6). The mRNA and protein levels of HSP47 and ECM-related markers were evaluated by (**A**) real-time PCR and (**B**) western blotting. Data are expressed as the mean ± SEM of three independent experiments. *p < 0.05 vs. control; ^†^p < 0.05 vs. TGF-β1.

To assess whether glucocorticoids suppressed TGF-β1-induced HSP47 and ECM expression in nasal fibroblasts, we pretreated cells with dexamethasone (2.5 μM) or fluticasone propionate (2.5 μM) for 1 h and then stimulated them with TGF-β1. Glucocorticoids decreased TGF-β1-induced HSP47, α-SMA, fibronectin, and collagen type I mRNA and protein expression (Fig. 6A,B).

### Glucocorticoid reversed the stimulatory effects of TGF-β1 on HSP47 expression and ECM production in *ex vivo* organ cultures of the nasal inferior turbinate

**Figure 7:**
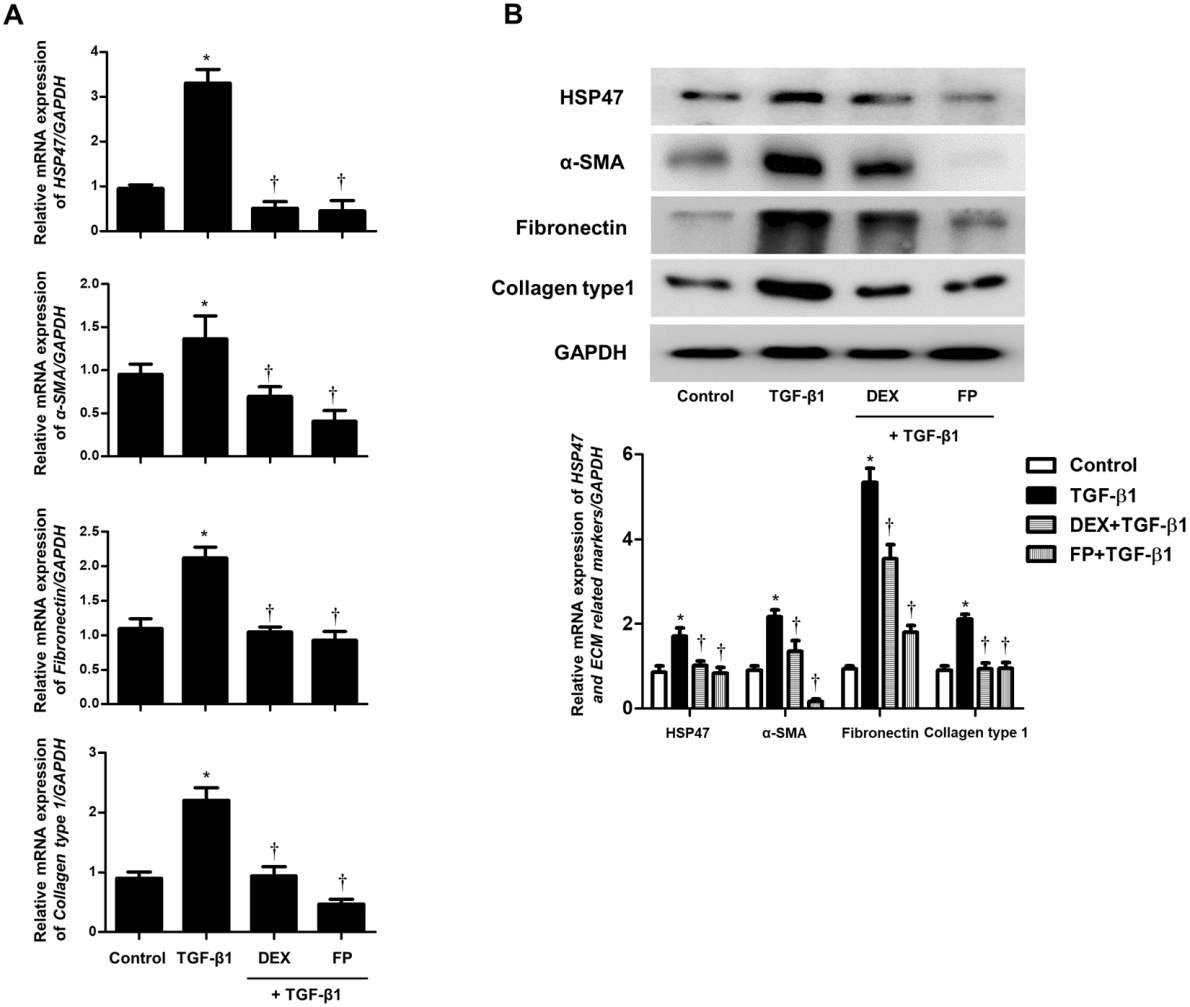
Glucocorticoids reduce TGF-β1-induced HSP47 and extracellular matrix expression in *ex vivo* organ cultures of nasal inferior turbinates. Nasal inferior turbinate tissues were organ cultured with or without dexamethasone (5 μM) and fluticasone propionate (5 μM) and then treated with TGF-β1 (2 ng/mL) in *ex vivo* organ cultures of nasal inferior turbinates (n = 6). (**A**) The mRNA levels for *HSP47* and ECM-related markers were evaluated using real-time PCR. (**B**) Shown are representative western blots for HSP47 and ECM-related markers. Data are expressed as the mean ± SEM of three independent experiments. *p < 0.05 vs. control; ^†^p < 0.05 vs. TGF-β1.

To determine the effect of glucocorticoids on HSP47, α-SMA, and ECM expression in nasal inferior turbinate tissue, we cultured *ex vivo* nasal inferior turbinates. Expression of HSP47, α-SMA, fibronectin, and collagen type 1 increased with TGF-β1 and was suppressed by glucocorticoids in both mRNA and protein levels (Fig. 7A,B). These results suggested that glucocorticoids could prevent ECM-associated protein expression by blocking HSP47 signaling pathways in sinonasal tissue in the presence of TGF-β1.

## Discussion

In the present study, we confirmed that HSP47 is increased in patients with CRSsNP. We tested which stimuli induced HSP47 expression in nasal fibroblasts, and we confirmed that TGF-β1 induced HSP47 expression in nasal fibroblasts. Myofibroblast differentiation and ECM production, which are induced by TGF-β1, were inhibited by HSP47 knockout using siRNA. We also confirmed that the Smad2/3 signaling pathway is involved in TGF-β1-induced HSP47 expression in nasal fibroblasts. In a functional assay including cell migration and collagen gel contraction assays, TGF-β1-enhanced migration and contraction ability were inhibited by HSP47 knockout. We showed that the use of glucocorticoids, a primary modality in CRS treatment, reversed the stimulatory effects of TGF-β1 on HSP47 expression and ECM production in nasal fibroblasts. We also confirmed the same effects by glucocorticoids on HSP47 expression and ECM production in *ex vivo* organ cultures of the nasal inferior turbinate.

Although there have been many studies on CRS, little is known about CRS pathogenesis. This is because CRS is a heterogeneous disease with varying pathophysiology depending on endotype and phenotype. Overall, CRS is divided into two groups according to the absence or presence of nasal polyps: CRSsNP and CRSwNP, respectively. CRSwNP is characterized morphologically by edema, goblet cell hyperplasia of the epithelium, lack of collagen production within the ECM, and abundant leukocytes, predominantly eosinophils. CRSsNP is also characterized by basement membrane thickening and goblet cell hyperplasia; however, the main inflammatory cells observed in CRSsNP are neutrophils. Several chemokines, including TGF-β and CXCL8, play a role in CRSsNP remodeling, which causes ECM deposition^15^. TGF-β1 is a well-known profibrotic cytokine. In a previous study, we showed that TGF-β1 stimulates myofibroblast differentiation of nasal fibroblasts, which is a major biological mechanism involved in tissue remodeling^16^. Moreover, TGF-β1 has been known to be increased in CRSsNP^17,18^. Among the several cytokines tested in this study, we propose that TGF-β1 induces HSP47 expression in nasal fibroblasts.

HSP47 is a heat‐shock protein that interacts transiently with procollagen during its folding, assembly, and transport from the endoplasmic reticulum (ER) of mammalian cells. HSP47 is known to act as a molecular chaperone that facilitates the folding and assembly of procollagen molecules, to retain unfolded molecules within the ER, and to assist in the transport of correctly folded molecules from the ER to the Golgi apparatus^19^. Although the exact mechanisms of pathologic tissue remodeling in airway diseases including CRS are not fully understood, increased production and deposition of collagens are characteristic findings observed in such diseases, as they are the result of a final common pathway in tissue remodeling. When we consider the essential role of HSP47 in collagen maturation, it is unsurprising that increased HSP47 expression is consistently observed in human fibrotic diseases that show excessive accumulation of collagen in their histopathology. In lower airways, increased HSP47 expression leads to an excessive accumulation of collagen in human pulmonary fibrosis disease in *in vivo* experiments^11,20^. Here, we showed that HSP47 is also increased in upper airway chronic inflammatory disease. We compared the expression patterns of HSP47 by dividing sinonasal tissue into four types: normal–UP, CRSsNP-UP, CRSwNP-UP, and CRSwNP-NP. We observed that HSP47 is increased in CRSsNP relative to the normal healthy control and CRSwNP patients. We also examined the relationship between HSP47 and ECM production in nasal fibroblasts. We clearly demonstrated that HSP47 is involved in ECM accumulation, which is induced by TGF-β1 in nasal fibroblasts. These results suggest that HSP47 is associated with tissue remodeling in CRSsNP.

TGF-β isoforms that activate the Smad2/3 intracellular pathway have been heavily implicated in fibrosis. Among them, TGF-β1 is considered a major driver of human fibrotic pathologies^21^. Accordingly, TGF-β1/Smad2/3 signaling is currently being explored as a target for the reduction of pathologic tissue remodeling. We have previously confirmed that TGF-β1 induces myofibroblast differentiation and extracellular production via the Smad2/3 signaling pathway in nasal fibroblasts^22^. Here, we showed that HSP47 knockout by siRNA did not inhibit the phosphorylation of Smad2/3, but inhibition of Smad2/3 phosphorylation by SIS3 inhibited HSP47 expression in TGF-β1-stimulated nasal fibroblasts. These results indicated that HSP47 contributes to the ECM process downstream of the TGF-β1/Smad2/3 pathway.

Glucocorticoids, such as dexamethasone and fluticasone propionate, are highly effective at alleviating nasal symptoms and improving nasal airflow, so they are used as the first line of treatment in the form of a spray for several airway inflammatory diseases, including CRS^23^. Accordingly, understanding the molecular mechanisms underlying the biological and pharmacological effects of glucocorticoids in CRS is necessary for the treatment of CRS patients. Generally, glucocorticoids are considered to be powerful modulators of inflammatory diseases. However, recent evidence showed that glucocorticoids may affect not only the prevention of inflammation, but also the inhibition of tissue remodeling in CRS. In the present study, we showed that glucocorticoids inhibited TGF-β1-induced HSP47 expression, and subsequently, ECM production in nasal fibroblasts. By combining this with our previously reported results that glucocorticoids modulated TGF-β1-induced Epithelial-mesenchymal transition processes related to tissue remodeling in nasal epithelial cells, we can assume that the anti-fibrotic effect exhibited by steroids could be an important therapeutic mechanism in glucocorticoids in CRS treatment^24^.

The data produced in this study suggest that TGF-β1-induced HSP47 regulates ECM accumulation in nasal fibroblasts. HSP47 siRNA attenuated TGF-β1-induced ECM production in nasal fibroblast cells, suggesting that HSP47 is involved in regulating ECM synthesis. Additionally, the Smad2/3 signaling pathways are involved in the enhancement of HSP47 expression by TGF-β1 in nasal fibroblasts. We described a mechanism by which dexamethasone and fluticasone propionate exerted anti-ECM activities. Limitation of our study are (1) we classified by phenotypes instead of classifying by immunologic features such as the eosinophilic and non-eosinophilic CRS, (2) we did not checked whether inhibition of HSP47 cause aggravation of CRS *in vivo*, (3) siRNA has disadvantages, such as the variability and incompleteness of knockdowns. Our results suggested that HSP47 could be a new therapeutic target for CRS treatment, and this may serve as a basis for a therapeutic agent that could replace glucocorticoids.

## Supplementary information

Supplementary information
