# Author Correction: TGF-β1-induced HSP47 regulates extracellular matrix accumulation via Smad2/3 signaling pathways in nasal fibroblasts

**DOI:** 10.1038/s41598-020-66547-z

**Published:** 2020-06-09

**Authors:** Hae-Ji Kim, Joo-Hoo Park, Jae-Min Shin, Hyun-Woo Yang, Heung-Man Lee, Il-Ho Park

**Affiliations:** 1 Upper Airway Chronic inflammatory Diseases Laboratory, Korea University, College of Medicine, Seoul, Korea; 2 Medical Devices Clinical Trials Laboratory, Korea University, College of Medicine, Seoul, Korea; 3 IVD Support Center Korea University, Korea University, College of Medicine, Seoul, Korea; 4 Department of Otorhinolaryngology-Head and Neck Surgery, Korea University, College of Medicine, Seoul, Korea

Correction to: *Scientific Reports* 10.1038/s41598-019-52064-1, published online 29 October 2019

This Article contains errors.

As a result of an error during figure assembly, some of the HSP47/DAPI images in Figure 2F duplicate HSP47/DAPI images in Figure 3C. Specifically, the control image in Figure 2F duplicates the first HSP47/DAPI image in Figure 3C, the 0.25 +TGF-β1 image in Figure 2F duplicates the third HSP47/DAPI image in Figure 3C, and the 0.75 +TGF- β 1 image in Figure 2F duplicates the second HSP47/DAPI image in Figure 3C. The correct Figure 2F is shown below as Figure 1.

**Figure 1:**
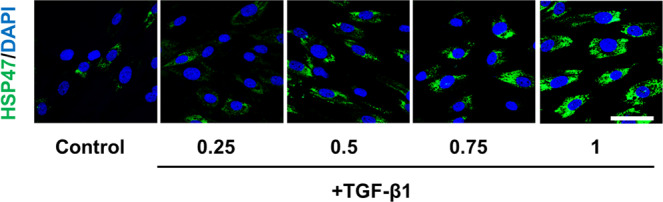
.

Original data for the Western blots shown in the main figures was not included in the Article. It is now included in the Supplementary Information file attached below.

These corrections do not affect the overall conclusions of the Article.

## Supplementary information

Supplementary information.

