# Supplementary material for: TGF-β1-induced HSP47 regulates extracellular matrix accumulation via Smad2/3 signaling pathways in nasal fibroblasts
Source: Sci Rep. 2019 Oct 29;9:15563. doi: 10.1038/s41598-019-52064-1 (PMC6820875; doi:10.1038/s41598-019-52064-1)
Supplement: Supplementary file 1 — Supplementary information [file 41598_2019_52064_MOESM1_ESM.docx]

**TGF-β1-induced HSP47 regulates extracellular matrix accumulation via Smad2/3 signaling pathways in nasal fibroblasts**

Hae-Ji Kim, MS^1§^, Joo-Hoo Park, PhD^1,2,4§^, Jae-Min Shin, MD^1, 2, 4^, Hyun-Woo Yang, MS^1,4^, Heung-Man Lee, MD, PhD^1, 2, 4†^, Il-Ho Park, MD, PhD^1, 2, 3, 4†^

^1^Upper Airway Chronic inflammatory Diseases Laboratory, Korea University, College of Medicine, Seoul, Korea; ^2^Department of Otorhinolaryngology-Head and Neck Surgery, Korea University, College of Medicine, Seoul, Korea; ^3^IVD Support Center, Korea University, College of Medicine, Seoul, Korea; ^4^ Medical Devices Clinical Trials Laboratory, Korea University, College of Medicine, Seoul, Korea.

^§^ These authors contributed equally to this work

Corresponding authors:

Il-Ho Park

Department of Otorhinolaryngology–Head and Neck Surgery,

Guro Hospital, Korea University College of Medicine,

80 Guro-dong, Guro-gu, Seoul 152-703, South Korea

Telephone: 82-2-2626-1298

Fax: 82-2-868-0475

E-mail: parkil5@korea.ac.kr

Heung-Man Lee

Department of Otorhinolaryngology–Head and Neck Surgery,

Guro Hospital, Korea University College of Medicine,

80 Guro-dong, Guro-gu, Seoul 152-703, South Korea

Telephone: 82-2-2626-3185

Fax: 82-2-868-0475

E-mail: [lhman@korea.ac.kr](mailto:lhman@korea.ac.kr)

**Supplementary information**

**Supplementary Table S1.** Clinical characteristics of patients (N=37)

| **Characteristics** | **Healthy UP**  **(n=4)** | **CRSsNP-UP**  **(n=10)** | **CRSwNP-UP (n=10)** | **CRSwNP-NP (n=13)** |
| --- | --- | --- | --- | --- |
| **No. women/men** | 3/1 | 3/7 | 2/8 | 4/9 |
| **Age, y, mean±SD** | 40.3±5.7 | 46.6±4.2 | 44.6±6.7 | 49.3±7.1 |
| **Asthma** | 0 | 0 | 0 | 0 |
| **Allergic rhinitis** | 0 | 0 | 0 | 0 |
| **Lund-Mackay**  **CT score** | 0.75 | 12.9 | 16.2 | 15 |
| ***UP, uncinate process; NP, nasal polyps; CRSsNP, chronic rhinosinusitis without nasal polyps; CRSwNP, chronic rhinosinusitis with nasal polyps; SD, standard deviation.*** | | | | |

**Supplementary Table S2.** Sequences of PCR primers

| **Gene Name** |  | **Sequences (quantitative RT-PCR)** |
| --- | --- | --- |
| ***HSP47*** | Forward | 5ʹ-GCTGAAGATCTGGATGGGGAAG-3ʹ |
|  | Reverse | 5ʹ-CTTGTCAATGGCCTCAGTCAGG-3ʹ |
| ***α-SMA*** | Forward | 5ʹ-CTGTTCCAGCCATCCTTCAT-3ʹ |
|  | Reverse | 5ʹ-CCGTGATCTCCTTCTGCATT-3ʹ |
| ***Fibronectin*** | Forward | 5ʹ-GGATGCTCCTGCTGTCAC-3ʹ |
|  | Reverse | 5ʹ-CTGTTTGATCTGGACCTGCAG-3ʹ |
| ***Collagen type1*** | Forward | 5ʹ-CATCACCTACCACTGCAAGAAC-3ʹ |
|  | Reverse | 5ʹ-ACGTCGAAGCCGAATTCC-3ʹ |
| ***GAPDH*** | Forward | 5ʹ-GTGGATATTGTTGCCATCAATGACC-3ʹ |
|  | Reverse | 5ʹ-GCCCCAGCCTTCTCCATGGTGGT-3ʹ |
