# Supplementary material for: Author Correction: TGF-β1-induced HSP47 regulates extracellular matrix accumulation via Smad2/3 signaling pathways in nasal fibroblasts
Source: Sci Rep. 2020 Jun 9;10:9585. doi: 10.1038/s41598-020-66547-z (PMC7280506; doi:10.1038/s41598-020-66547-z)
Supplement: Supplementary file 1 — Supplementary information. [file 41598_2020_66547_MOESM1_ESM.docx]

**TGF-β1-induced HSP47 regulates extracellular matrix accumulation via Smad2/3 signaling pathways in nasal fibroblasts**

Hae-Ji Kim, MS^1§^, Joo-Hoo Park, PhD^1,2,4§^, Jae-Min Shin, MD^1, 2, 4^, Hyun-Woo Yang, MS^1,4^, Heung-Man Lee, MD, PhD^1, 2, 4†^, Il-Ho Park, MD, PhD^1, 2, 3, 4†^

^1^Upper Airway Chronic inflammatory Diseases Laboratory, Korea University, College of Medicine, Seoul, Korea; ^2^Medical Devices Clinical Trials Laboratory, Korea University, College of Medicine, Seoul, Korea.; ^3^IVD Support Center, Korea University, College of Medicine, Seoul, Korea; ^4^Department of Otorhinolaryngology-Head and Neck Surgery, Korea University, College of Medicine, Seoul, Korea

^§^ These authors contributed equally to this work

Corresponding authors:

Il-Ho Park

Department of Otorhinolaryngology–Head and Neck Surgery,

Guro Hospital, Korea University College of Medicine,

80 Guro-dong, Guro-gu, Seoul 152-703, South Korea

Telephone: 82-2-2626-1298

Fax: 82-2-868-0475

E-mail: parkil5@korea.ac.kr

Heung-Man Lee

Department of Otorhinolaryngology–Head and Neck Surgery,

Guro Hospital, Korea University College of Medicine,

80 Guro-dong, Guro-gu, Seoul 152-703, South Korea

Telephone: 82-2-2626-3185

Fax: 82-2-868-0475

E-mail: [lhman@korea.ac.kr](mailto:lhman@korea.ac.kr)

**Supplementary information**

**
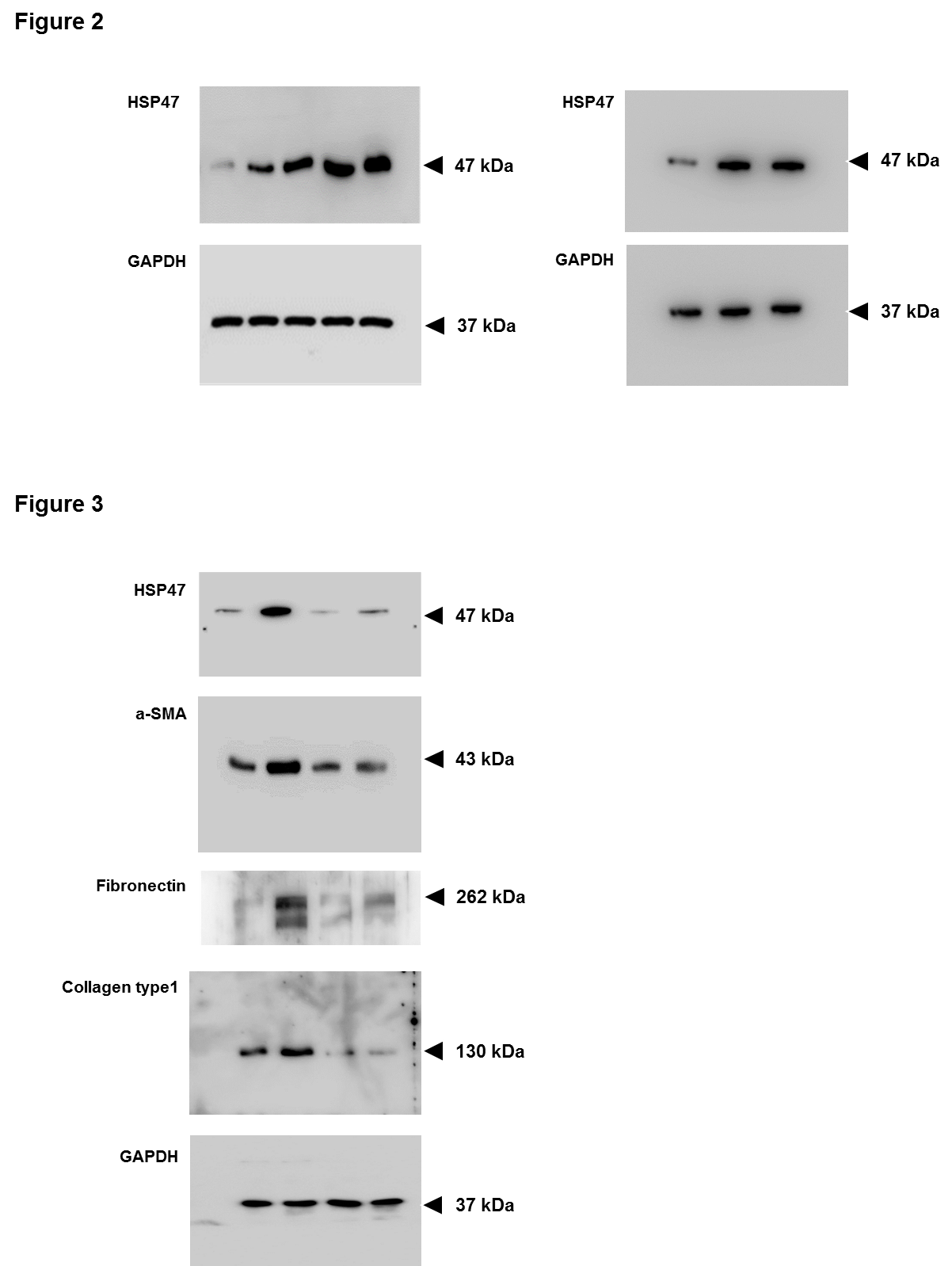
**

**
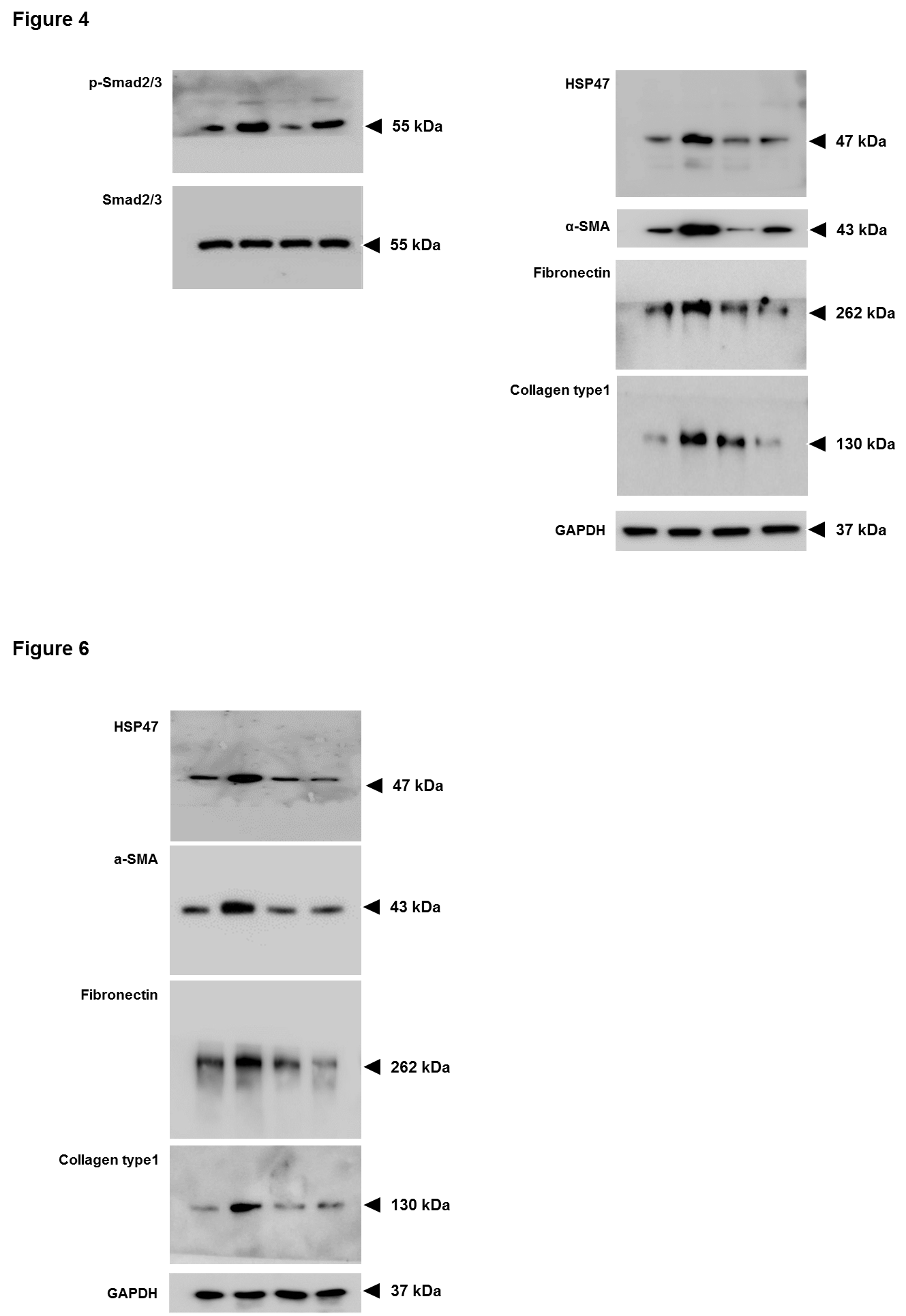
**

**
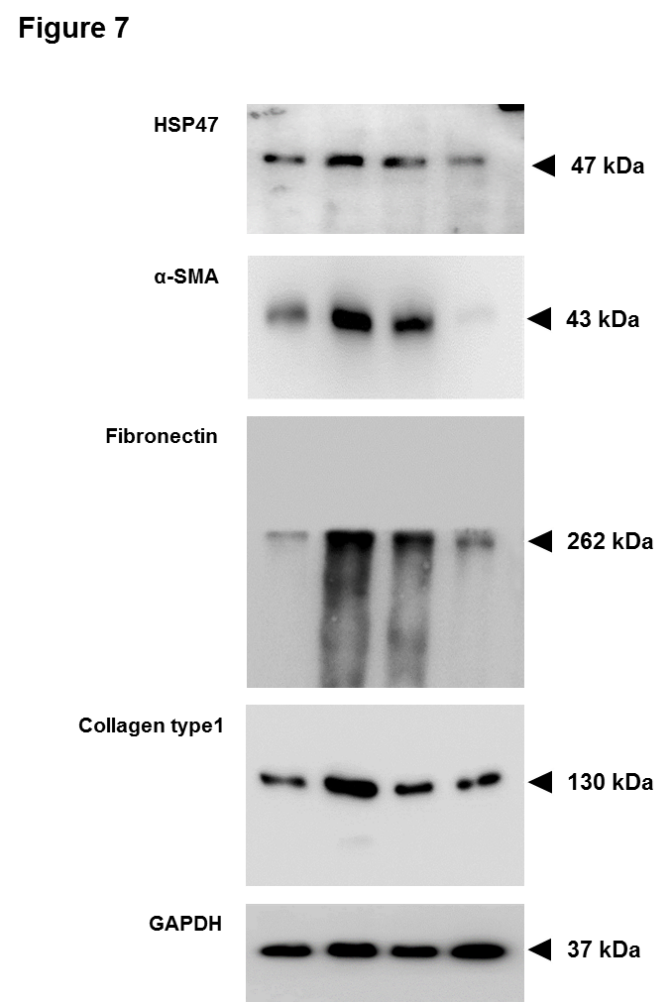
**
